# Practical issues and research trends of oncofertility in gynecologic cancer

**DOI:** 10.4069/kjwhn.2021.05.31

**Published:** 2021-06-17

**Authors:** Jeong-Yeol Park

**Affiliations:** Department of Obstetrics and Gynecology, Asan Medical Center, University of Ulsan College of Medicine, Seoul, Korea

**Keywords:** Endometrial neoplasma, Fertility preservationr, Ovarian neoplasms, Uterine cervical neoplasms

## Introduction

Cervical, endometrial, and ovarian cancer are the most common gynecologic cancers in South Korea (hereafter, Korea) [1]. Cervical cancer is the most common, followed by endometrial and ovarian cancer [1]. In a major change in the epidemiology of gynecologic cancer in Korea, the incidence of cervical cancer is rapidly decreasing and the incidence of endometrial cancer and ovarian cancer is rapidly increasing [2]. Notably, although the incidence of cervical cancer is decreasing overall, the incidence of cervical cancer and endometrial cancer is increasing among young women [2]. In addition, as the birth rate falls and the average age at which women first give birth gradually increases, the incidence of gynecologic cancer among young women who have not yet undergone childbirth is also increasing [3]. Therefore, there has been a greater impetus in recent years on preserving fertility in young women with gynecologic cancer. For young women who have not undergone childbirth, the preservation of fertility after treatment for gynecologic cancer has a significant impact on their quality of life. For early-stage gynecologic cancers, fertility-sparing treatment makes future pregnancy possible by treating the cancer and leaving parts of the uterus and ovaries intact. For advanced gynecologic cancers, treatments that leave the uterus and ovaries intact are not always possible, and radiation therapy or chemotherapy, which can lead to infertility, are often required. Therefore, assisted reproductive technology is required to preserve fertility. This article describes methods for preserving fertility in young women with gynecologic cancer and examines practical issues and research trends related to oncofertility in gynecologic cancer.

## Fertility-sparing treatment for young women with early-stage gynecologic cancer

The cure rate for stage I gynecologic cancers is very high, underscoring the need to focus on improving patients’ quality of life. Fertility preservation after gynecologic cancer treatment in young women who have not yet given birth to children is one of the major quality of life problems for this population. Guidelines for safe fertility-sparing management in women who want to become pregnant after treatment for gynecologic cancer have been established to some extent based on the results of numerous studies.

### Fertility-sparing treatment for young women with early-stage cervical cancer

Radical trachelectomy is a fertility-sparing surgical treatment for young women with early-stage cervical cancer who want to preserve fertility [4]. In this surgical procedure, the cervix, paracervical tissue, and upper vagina are removed, and the thin rim of the cervix and the uterine body are left to connect to the vagina [5]. The widely accepted criteria for considering radical trachelectomy are as follows: (1) the patient is a young woman who eagerly wants to preserve fertility; (2) the patient has stage I cervical cancer that is confined to the uterine cervix; (3) the tumor size is <2 cm; (4) squamous cell carcinoma, adenocarcinoma, or adenosquamous carcinoma is present, and (5) the patient shows no evidence of prior infertility [6]. Radical trachelectomy can be performed through vaginal, abdominal, laparoscopic, and robotic surgical approaches. The first procedure of this type that was performed was vaginal radical trachelectomy [5,7]. However, the procedure for the vaginal approach is difficult to learn, and the radicality of the operation is limited. To compensate for these shortcomings, an abdominal approach was developed [8]. Considering the advantages of minimally invasive surgery compared to open surgery, this procedure is now often performed using laparoscopic surgery or robotic surgery [9,10]. It was reported that the results of radical trachelectomy tend to be similar to those of radical hysterectomy when the indications for potential patients were adhered to properly [11,12].

According to a recent meta-analysis, there is no substantial difference between vaginal, abdominal, laparoscopic, and robotic radical trachelectomies in terms of oncological treatment outcomes and posttreatment pregnancy outcomes [13-15]. In cases of cervical cancer with tumors larger than 2 cm, research has been undertaken to determine if radical trachelectomy can be performed after the tumor decreases in size as a result of neoadjuvant chemotherapy [16].

In addition, other studies have examined the effectiveness of less radical surgical procedures such as conization or simple trachelectomy for cervical cancer cases where the tumor is small [17]. Research has also been performed to compare the abdominal approach and the minimally invasive approach for radical trachelectomy [18].

### Fertility-sparing treatment for young women with early-stage endometrial cancer

For endometrial cancer, fertility-sparing treatment primarily consists of hormone therapy [19,20]. Among the various types of hormone therapies, the most widely used and researched is treatment using progestin [19,20]. High-dose oral progestin such as medroxyprogesterone acetate and megestrol acetate or a progestin-releasing intrauterine device are the most common methods for administering progestin [19,20]. The widely accepted criteria for potential patients of fertility-sparing treatment in endometrial cancer are as follows: (1) the patient is a young woman who eagerly wants to preserve fertility; (2) the patient has stage I endometrial cancer confined to the endometrium; (3) endometrioid adenocarcinoma is present; and (4) the patient shows no prior evidence of infertility [21]. It is known that complete remission occurs in approximately 76% of cases treated with progestin therapy [22,23]. The remaining 24% of patients do not respond to progestin therapy and hysterectomy should be performed instead [22,23]. For patients with complete remission due to progestin therapy, attempting pregnancy immediately is recommended. Since the recurrence rate of endometrial cancer is very high, hysterectomy is recommended after patients in remission complete family planning [22,23]. The pregnancy outcomes after fertility-sparing treatment using progestin are promising [24]. Recent studies have examined a treatment strategy in which all endometrial cancer tissues are removed via hysteroscopic surgery and adjuvant hormone therapy is administered [19,25]. In addition, studies have examined the therapeutic effects of metformin, aromatase inhibitors, gonadotropin-releasing hormone agonists, and several drug combinations [26].

### Fertility-sparing treatment for young women with early-stage epithelial ovarian cancer

Epithelial ovarian cancer is the most common histological type of ovarian cancer. Fertility-sparing treatment for women with epithelial ovarian cancer involves preserving the uterus and part of the normal ovarian tissue during surgery to enable pregnancy after treatment [27]. The widely accepted criteria for potential patients with epithelial ovarian cancer to receive fertility-sparing surgery are as follows: (1) the patient is a young woman who eagerly wants to preserve fertility; (2) the patient has stage I epithelial ovarian cancer confined to one ovary; and (3) the patient shows no prior evidence of infertility [28]. In recent large studies, the survival outcomes after fertility-sparing surgery for stage I epithelial ovarian cancer patients were reported to be similar to those of patients who underwent standard surgical treatment [29,30]. Pregnancy outcomes were also reported to be good [27].

## Options to preserve fertility for young women with advanced-stage gynecologic cancer

Surgical treatment for advanced gynecologic cancer can lead to situations in which the uterus and/or ovaries cannot be preserved. In addition, infertility can occur after radiation therapy or gonadotoxic chemotherapy. In this situation, thorough consultation before treatment is required for patients who wish to preserve fertility, and assisted reproductive technology is required.

If the ovaries should be removed surgically or pelvic radiation therapy or gonadotoxic chemotherapy is planned, a strategy to collect and store the patient’s eggs is needed before treatment. These methods include embryo cryopreservation, oocyte cryopreservation, and ovarian tissue cryopreservation. Ovarian transposition can also be an option before pelvic radiation therapy. If the patient has a spouse, embryo cryopreservation is the most effective method [31]. However, it takes over 2 weeks for ovarian hyperstimulation, transvaginal ovum pick-up, and fertilization before cryopreservation of an embryo is possible [31]. In the absence of a spouse, oocyte cryopreservation can be performed [31]. Immediately after transvaginal ovum pick-up, the patient’s oocytes are frozen, but this process also takes over 2 weeks [31]. As of recently, ovarian tissue cryopreservation can also be performed [32]. One advantage of ovarian tissue cryopreservation is that no additional time is required to induce ovarian hyperstimulation, so there is no need to postpone anti-cancer treatment [32]. If these processes cannot be performed before treatment, ovum donation should be selected as the method for inducing pregnancy.

If the uterus has been removed, a surrogate mother can be used in family planning. The development of artificial uteruses and research in the field of uterine transplantation are in the beginning stages, and no conclusions can be drawn yet.

## Conclusion

For women with gynecologic cancers, there are several ways to preserve fertility and perform childbirth after treatment. For women with early-stage gynecologic cancers, one method of doing so is to preserve parts of the uterus and ovaries. If the uterus and ovaries cannot be preserved, or radiation therapy and gonadotoxic chemotherapy are required, assisted reproductive technology can be used as a method to preserve fertility. If a woman diagnosed with gynecologic cancer wishes to preserve fertility in order to give birth after treatment, it is necessary to discuss these treatment options in detail before treatment.
